# Docosahexaenoic Acid Protects Schwann Cells Against Palmitic Acid–Induced Lipotoxicity by Modulating Autophagy, ER Stress, and Lipid Handling

**DOI:** 10.1007/s11064-026-04841-4

**Published:** 2026-07-25

**Authors:** Francis Zamora, Jo-Wen Liu, Viet Hoang Dinh, Alfonso M. Duran, Marino A. De León, Johnny D. Figueroa

**Affiliations:** 1 Center for Health Disparities and Molecular Medicine, Department of Basic Sciences, Physiology Division, Department of Basic Sciences, Linda University Health School of Medicine, 11085 Campus Street, Mortensen Hall, Loma Linda, CA 92350 USA; 2 Department of Pathology and Human Anatomy, Loma Linda University Health School of Medicine, Loma Linda, CA USA

**Keywords:** Lipotoxicity, FABP5, Schwann cell, Autophagy, Docosahexaenoic acid (DHA)

## Abstract

Elevated saturated fatty acids, such as palmitic acid (PA), induce lipotoxicity in peripheral nerve cells, a pathological feature of metabolic disorders such as type 2 diabetes and obesity that are frequently associated with neuropathic pain (NP). PA overload elicits a maladaptive stress response characterized by endoplasmic reticulum (ER) stress, disrupted intracellular calcium homeostasis, and impaired autophagic flux, ultimately promoting cell death. Although omega-3 polyunsaturated fatty acids such as docosahexaenoic acid (DHA) protect against PA-induced lipotoxicity (PA-LTx), the mechanisms linking lipid handling, ER stress, and autophagy in Schwann cells remain poorly defined. Here, we investigated how PA and DHA regulate autophagic flux, ER stress signaling, and fatty acid–binding protein 5 (FABP5)–dependent lipid trafficking in immortalized Schwann cells (ISCs). PA exposure (300 µM PA:150 µM BSA, 24–48 h) significantly reduced cell viability, impaired autophagic flux as indicated by LC3-II and p62 accumulation, disrupted autophagosome–autolysosome balance, and increased susceptibility to autophagic inhibition by chloroquine. DHA co-treatment (50 µM) preserved cell viability, restored autophagic flux, and normalized autophagosome–autolysosome fusion. Mechanistically, PA induced ER stress marked by increased CHOP, ATF4, and Xbp1 expression, along with progressive ER calcium depletion, whereas DHA suppressed these responses and stabilized calcium homeostasis. Building on prior evidence that FABP5 protects neuron-like cells from PA-LTx, we identified a regulatory role for FABP5 in Schwann cells. PA robustly induced FABP5 expression, which was normalized by DHA and modulated by pharmacological manipulation of autophagy. FABP5 silencing exacerbated PA-induced ER stress, triggered a dysfunctional compensatory autophagy response, and impaired DHA-induced lipid droplet formation. Collectively, these findings demonstrate that functional autophagy and FABP5-dependent lipid buffering are critical adaptive responses to lipotoxic stress in Schwann cells, highlighting these pathways as potential therapeutic targets for NP-associated metabolic neuropathies.

## Highlights

Palmitic acid impairs autophagic flux and reduces Schwann cell viability, effects that are exacerbated by pharmacological inhibition of autophagy.

Docosahexaenoic acid preserves Schwann cell survival during lipotoxic stress by restoring autophagic flux and autophagosome–autolysosome maturation.

DHA attenuates palmitic acid–induced ER stress signaling and stabilizes intracellular calcium homeostasis in Schwann cells.

FABP5 expression is regulated by lipotoxic stress and autophagy modulation, linking lipid handling to cellular stress responses.

FABP5 is required for DHA-induced lipid droplet formation and supports adaptive autophagy and ER stress regulation during lipotoxic conditions.

## Introduction

Neuropathic pain (NP) is a chronic and disabling condition characterized by aberrant sensory processing, including allodynia, hyperalgesia, and paresthesia [1]. NP is highly prevalent in metabolic disorders such as type 2 diabetes, obesity, and metabolic syndrome [2], conditions frequently accompanied by dyslipidemia and chronically elevated circulating non-esterified fatty acids. Although hyperglycemia and microvascular dysfunction are established contributors to metabolic stress-induced neuropathy, accumulating evidence supports a pathophysiological role for lipid overload as an additional driver of peripheral nerve injury [3, 4].

Chronic exposure to saturated fatty acids, particularly palmitic acid (PA), induces lipotoxic stress by overwhelming cellular lipid-handling capacity, leading to organelle dysfunction and cell death [5–7]. Diets enriched in saturated fat increase pain-like behaviors in rodents even in the absence of overt nerve injury or obesity [8–10], supporting a role for lipid stress in NP-relevant phenotypes. At the cellular level, PA disrupts endoplasmic reticulum (ER) homeostasis, mitochondrial function, reactive oxygen species accumulation, and lysosomal integrity in neural systems [3, 5, 6, 11].

Docosahexaenoic acid (DHA), a long-chain omega-3 polyunsaturated fatty acid, is a major structural component of the nervous system, supporting membrane fluidity and modulating inflammatory and cellular stress responses [12–14]. Experimental studies from our group and others have shown that DHA protects neural cells against PA-induced lipotoxicity by promoting autophagy, reducing apoptotic and necroptotic signaling, and activating pro-survival pathways, including AKT–mTORC2 signaling [15, 16]. In a recent clinical study, we demonstrated that DHA-rich dietary supplementation in patients with type 2 diabetes and neuropathic pain reduced circulating neurotoxic lipid mediators and was accompanied by changes in lipid-handling and autophagy-associated markers [17–19]. These findings provide in vivo evidence that DHA modulates lipid stress pathways relevant to neuropathic pain. However, how DHA modulates lipid-handling pathways in Schwann cells—metabolically active glial cells essential for peripheral nerve function—under lipotoxic conditions remains unresolved.

A unifying mechanistic framework linking these observations centers on intracellular lipid-handling pathways. Unlike saturated fatty acids, DHA is preferentially esterified into neutral lipid pools and can promote lipid droplet formation, an adaptive response that buffers excess fatty acids and limits ER stress [20–22]. Lipid droplets also intersect with autophagy, contributing to ER homeostasis and providing lipid substrates for autophagosome membrane expansion [23–26]. Disruption of these processes can impair autophagic flux and exacerbate lipotoxic injury [27–29]. Together, these pathways provide a mechanistic framework for interpreting DHA-driven changes in lipid stress and autophagy markers observed in clinical studies; however, their relevance in peripheral nerve cells remains unknown.

Autophagy plays an important role in peripheral nerve biology, including Schwann cell-mediated myelin turnover and nerve repair [30, 31]. Schwann cell autophagy has also been implicated in modulating neuropathic pain and peripheral nerve resilience [32–35]. Autophagic dysfunction is a feature of metabolic and diabetic neuropathy models and restoring autophagy can improve cellular and functional outcomes [36, 37, 38–40]. However, It remains unclear whether lipid-induced autophagy impairment in Schwann cells underlies the DHA-responsive signatures observed in human neuropathy.

Fatty acid binding protein 5 (FABP5) is a stress-responsive lipid chaperone expressed in neural tissues. FABP5 contributes to lipid-mediated adaptive responses following injury and metabolic stress [41–43] and supports neural remodeling processes during development and recovery [44–46]. Emerging evidence further implicates FABP5 in lipid droplet dynamics, metabolic adaptation, and autophagy-associated pathways [47–50, 82]. However, its role in Schwann cells and in coordinating lipid stress-induced autophagy remains poorly defined.

Building on unresolved mechanistic questions raised by prior clinical observations, we tested the hypothesis that DHA preserves Schwann cell viability during PA-induced lipotoxicity by maintaining ER homeostasis and functional autophagic flux, with FABP5 contributing to these adaptive responses through regulation of intracellular trafficking. Using an immortalized Schwann cell model, we directly interrogate the interplay among lipid stress, ER signaling, autophagy dynamics, lipid droplet formation, and FABP5 function, thereby providing mechanistic insight into the cellular pathways that may underlie the benefits of DHA in metabolic stress-associated neuropathy.

## Materials and Methods

### Cell Culture and Fatty Acid Treatment

The immortalized Schwann cell line (ISCs) used in this study was a gift from Dr. Laurel Bolin [51] and has been shown to be comparable to primary Schwann cell cultures, as evidenced by similar expression of specific SC markers. Cells were cultured in poly-L-lysine-coated flasks and maintained in full serum media consisting of Dulbecco’s modified Eagle’s medium/Ham’s F12 (DMEM/F12) 50/50 mix without glutamine (Mediatech, Herndon, VA), supplemented with 10% horse serum (Invitrogen, Carlsbad, CA), 1% L-glutamine (Mediatech), and 1% penicillin/streptomycin (Mediatech). ISC cultures were incubated at 37 °C in 5% CO2, with the media replaced every two days. Cells were used at ≤ 5 passages for all experiments to ensure phenotypic stability and reproducibility.

For experimental treatments, cells were maintained in serum-free media consisting of DMEM/F12 (50/50), 1% L-glutamine, 1% penicillin/streptomycin and 1X N-2 supplement (Gibco, Life Technologies Corp., Grand Island, NY). The inclusion of N-2 supplement supports cell viability under serum-free conditions while limiting proliferation of iSCs.

Cells were initially plated on poly-L-lysine-coated plates in full serum media and allowed to attach overnight. Full serum media was then removed, the cells were washed once with Dulbecco’s Phosphate-Buffered Saline (DPBS), and then replaced with serum-free media for at least 12 h prior to treatment.

For palmitic acid-induced lipotoxicity (PA-LTx) experiments, cells were treated with a palmitic acid (PA): bovine serum albumin (BSA) (2:1) complex as described previously [3, 5]. Briefly, a 300 mM PA (Sigma-Aldrich, St. Louis, MO) stock was prepared by dissolving PA in 100% ethanol. PA-containing media was prepared by dissolving fatty-acid-free BSA (EMD Biosciences, San Diego, CA) into serum-free media at 150 µM and warming to 37 °C. PA stock was added to warm media to a final treatment concentration of 300 µM (final ethanol concentration, 0.1%), and the mixture was vortexed for 2 min to ensure complete dissolution. The PA-prepared media was placed in the incubator overnight and vortexed for an additional 2 min prior to treatments to ensure proper dissolution of PA. For cell treatments using docosahexaenoic acid (DHA) (Sigma-Aldrich), a 150 mM DHA stock was prepared in 100% ethanol and added directly to the treatment media to achieve a final concentration of 50 µM (DHA: BSA, 1:3, final ethanol concentration, 0.03%). For the control condition, serum-free media containing 150 µM BSA and 0.13% ethanol were prepared and warmed in a 37 °C water bath for 30 min prior to treatments.

Treatment concentrations were selected based on dose–response experiments and prior studies from our group (Supplementary Figure S1). PA at 300 µM reproducibly induces significant lipotoxicity and cell death [5, 15, 16], therefore was used as the lipotoxic stress condition throughout the study. In our cell line, DHA at 50 µM consistently provided maximal protection against PA-induced cytotoxicity at 48 h and was selected for subsequent experiments (Supplementary Figure S1).

Cells were treated with PA, DHA, or PA/DHA co-treatment for 24–48 h. Following treatments, morphological changes were observed, and images were captured at 40x on an Olympus microscope equipped with Hoffman modulation contrast (Olympus American) and a digital spot Imaging system (Diagnostic Instruments, Sterling Heights, MI).

### Cell Viability Assay

For cell viability assessment, cells were seeded at a density of 5,000 cells per well in 96-well poly-L-lysine-coated tissue culture plates. After 24- and 48-h treatments, WST-1 reagent (Roche Diagnostics, Indianapolis, IN, USA) diluted 1:10 in culture media was added directly to each well and incubated for 1.5 h at 37 °C according to the manufacturer’s instructions. Absorbance was measured at 440 nm using a SpectraMax i3X microplate reader (Molecular Devices, Sunnyvale, CA). Cell viability was assessed using 4 technical replicates per condition; values from technical replicates were averaged and expressed as a percentage relative to the control condition. Extra wells containing WST-1-prepared media without cells were used as blanks.

### [Ca^2+^]_ER_ Measurement

The Fluo-4 NW Calcium Assay Kit (Invitrogen) was used to assess ER calcium content after exposure of ISCs to PA, DHA, and PA/DHA co-treatment. Cells were seeded at 5,000 cells per well in 96-well poly-L-lysine-coated plates. After reaching the desired confluency and undergoing serum-free starvation, cells were treated for 30 min, 1 h, 3 h, and 24 h. At each time point, the treatment media were removed, and 100 µl of dye-loading solution containing 2.5 mM Probenecid was added to each well without washing. Cells were incubated for 30 min at 37 °C, followed by 30 min at room temperature. To selectively deplete ER calcium stores, 1 µM Thapsigargin or DMSO (vehicle control) was added directly to the wells during the final 2 min of the room-temperature incubation, and the resulting Thapsigargin-evoked Ca²⁺ release was used as a functional proxy for ER Ca²⁺ content. Fluorescence was immediately measured using the SpectraMax i3X microplate reader (Molecular Devices, Sunnyvale, CA) at 494 nm excitation and 516 nm emission. ER calcium content was calculated using 3 technical replications and expressed as fold change relative to the control condition.

### Western Blot Analysis

Following treatments, cells were lysed in Laemmli lysis buffer (0.1 M Tris-HCl, pH 6.8, containing 4% SDS, 10% glycerol, and a protease/phosphatase inhibitor cocktail). Following protein quantification, equal amounts of protein (25–30 µg) were resolved on 4–12% NuPAGE Bis-Tris gels (Invitrogen) and electrophoretically transferred to nitrocellulose membranes. Membranes were blocked for 30 min at room temperature with Intercept (TBS) Blocking Buffer (Li-Cor Biosciences, Lincoln, NE). To assess autophagy flux, membranes were incubated overnight at 4 °C with primary monoclonal antibodies against LC3A/B (Cell Signaling Technology, Cat# 4108 S) and Anti-SQSTM1/p62 (Abcam, Cat# EPR4844), both diluted in Intercept T20 (TBS) Antibody Diluent (Li-Cor Biosciences). Membranes were also probed with FABP5 (rabbit polyclonal antibody produced in the laboratory; [46])and C/EBP homologous protein (CHOP) (Cell Signaling Technologies, Cat#2895). Following washes with Tris-buffered saline containing 0.1% Tween 20 (TBST), membranes were incubated with appropriate secondary antibodies (goat anti-rabbit IRDye‐800 or goat anti-mouse IRDye-800; Li‐Cor Biosciences) for 1.5 h at room temperature. To verify equal protein loading, membranes were probed with β‐actin (clone AC‐15; Sigma‐Aldrich) and then with anti-mouse IRDye-680RD (Li‐Cor Biosciences). The Odyssey Infrared imager system (Li‐Cor Biosciences) was used to detect protein band intensity, and quantification was performed using Image Studio Software (Version 5.5; Li-COR Biosciences).

### Fluorescence Microscopy

ISCs were seeded onto 12-mm poly-L-lysine-coated glass coverslips (Corning, Cat# 354085) in 24-well plates at a density of 25,000 cells per coverslip and maintained overnight in full-serum media to allow cell attachment. To monitor autophagosome and autolysosome formation, full-serum media was replaced with serum-free media, and cells were transduced with a baculovirus expressing RFP-GFP-LC3B (Premo Autophagy Sensor RFP-GFP-LC3B kit, Molecular Probes, Carlsbad, CA) at a ratio of 4 µl per 10,000 cells for 18 h to allow sufficient reporter expression. Following transduction, cells were exposed to PA, DHA, and PA/DHA treatments for 24 h. Cells were then washed with PBS and gently fixed with 4% paraformaldehyde for 10 min. Fixed cells were immediately imaged using a Zeiss LSM 900 confocal microscope (Carl Zeiss Microscopy) equipped with a 63x oil-immersion objective, and z-stacks were acquired using appropriate fluorochrome excitation wavelengths (488 nm for GFP and 561 nm for RFP). In separate experiments to assess lipid droplet formation, ISCs were similarly treated and fixed as described above and stained with 10 µM BODIPY 493/503 (Invitrogen) for 30 min. Cells were then counterstained with ProLong Gold Antifade Mountant with DAPI (Invitrogen) and imaged by confocal microscopy using GFP wavelength settings. Identical acquisition parameters were maintained across all experimental conditions, and 2–4 images were taken for each coverslip.

### Quantitative Real-Time qPCR

Following treatments, total cellular RNA was extracted using TRI reagent (Molecular Research Center, Cincinnati, OH, USA) and quantified by measuring OD at 260 nm. Subsequently, 0.8–1 ug of RNA was reverse transcribed into cDNA using the iScript cDNA synthesis kit (Bio-Rad Laboratories, Hercules, CA, USA). Real-time qPCR was then performed in triplicate on a CFX96 system (Bio-Rad Laboratories) using SYBR Green-based detection to quantify gene expression. β-actin served as the reference gene. The 2^-ΔΔC_T_ method was used to calculate the relative mRNA abundance under our experimental conditions. The following primer sets were used in this study:

**Table:** 

Gene	FW	RV
Actin	GGGAAATCGTGCGTGACATT	GCGGCAGTGGCCATCTC
Atg5	TGTCTCTGCTGTCCTGTTGG	GCAGCGAACTTCCCTTACTG
Atg7	CCCAAAGACATCAAGGGCTA	CCTGACTTTATGGCTTCCCA
Atg12	CGTCTTCGGTTGCAGTTTC	CCAGTTTACCATCACTGCCA
P62	TCCGTACCTAGTCTGCGGTT	AGCTATCAGAGAGGTGGCCA
Chop	CAGCGACAGAGCCAAAATAA	TCCTCATACCAGGCTTCCAG
Atf4	GTTGGTCAGTGCCTCAGACA	CATTCGAAACAGAGCATCGA
Xbp1	CCCCCAAGTGCTACTCCTA	TTTCTATCTCGCGCAGTCTGT
Beclin1	TTCAAGATCCTGGACCGAGTGAC	AGACACCATCCTGGCGAGTTTC
Hsp70	GATGATCCGCAGCACGTTCAGA	AACTACAAGGGCGAGAACCGGTC
Hsp90	GGTTAGTCACGTTTCGTGCG	ATCCAGAGCGTCTGAGGAGT
Fabp5	TTACCCTCGACGGCAACAA	CCATCAGCTGTGGTTTCATCA

### ImageJ Quantification

All image processing and analysis were conducted by blinded investigators utilizing FIJI/ImageJ (Version 2.16.0/1.54p). For autophagy flux experiments using the RFP-GFP LC3 reporter, puncta were quantified using the Analyze Particles function after background subtraction. A fixed-intensity threshold was applied independently to the C1 (RFP) and C2 (GFP) channels, and the resulting binary masks were generated. LC3 puncta were quantified using size filters of 0.1–2.5 μm² and a circularity range of 0.3–1.0 to exclude background noise. To quantify colocalized (yellow) puncta, the C1 and C2 binary masks were combined using the Image Calculator AND operation, and the resulting merged mask was analyzed using the same particle size and circularity parameters. For total cell area measurements, a Mean auto-threshold was applied separately to the C1 channel, converted to a binary mask, and used to measure total area. Puncta count (C1, C2, yellow) were normalized to total cell area.

For lipid droplet quantification, BODIPY 493/503-stained images were background-subtracted and fixed intensity thresholded. DAPI fluorescence was quantified in parallel using a fixed intensity threshold, and lipid droplet integrated density was normalized to the corresponding DAPI signal. All image processing, thresholding, size constraints, and measurement parameters were kept constant across all experimental conditions and independent experiments.

### siRNA Transfection

To knock down FABP5, ISCs were transfected with Dicer-substrate siRNA (DsiRNA) duplexes targeting rat Fabp5 (IDT reference #487059275; duplex name: rn.Ri.Fabp5.13.2). The DsiRNA consisted of the following sequences:

Sense strand: 5’-rGrArCrUrUrUrUrCrArUrCrArUrArGrArCrArCrUrUrUrACC-3’.

Antisense strand: 5’-rGrGrUrArArArGrUrGrUrCrUrArUrGrArUrGrArArArArGrUrCrArU-3’.

A scrambled DsiRNA sequence was used as a negative control. Transfections were performed using INTERFERin transfection reagent (Polyplus, catalog #101000028) according to the manufacturer’s instructions. One day prior to transfection, ISCs were seeded in culture plates to reach 60–70% confluency at the time of transfection. On the day of transfection, full-serum media were replaced with antibiotic-free, serum-free medium. DsiRNA-INTERFERin complexes were prepared and added directly to the wells. Cells were incubated for 24 h post-transfection. Knockdown efficiency of FABP5 mRNA and protein levels was subsequently validated by real-time qPCR and Western blotting.

### Statistical Analysis

All experiments were performed independently at least three times and each experiment included technical replicates, as specified in the corresponding Methods sections, to ensure rigor and reproducibility. Data are presented as mean ± standard deviation (SD). Statistical analyses and graphs were conducted using GraphPad Prism (Version 10, GraphPad Software, San Diego, CA, USA). Multiple-group comparisons were made using one-way or two-way ANOVA with appropriate post hoc tests. Statistical significance was set at *p* < 0.05.

## Results

### Functional Autophagy Supports Cell Survival During Palmitic Acid-Induced Lipotoxicity and Contributes to DHA-Mediated Protection

To assess the role of autophagic flux during PA-LTx, ISCs were exposed to 300 µM PA, 50 µM DHA, and PA/DHA co-treatments in the presence or absence of 20µM chloroquine (CQ), an inhibitor of autophagic flux, for 24 and 48 h. Cell viability was measured using the WST-1 assay.

**Fig. 1:**
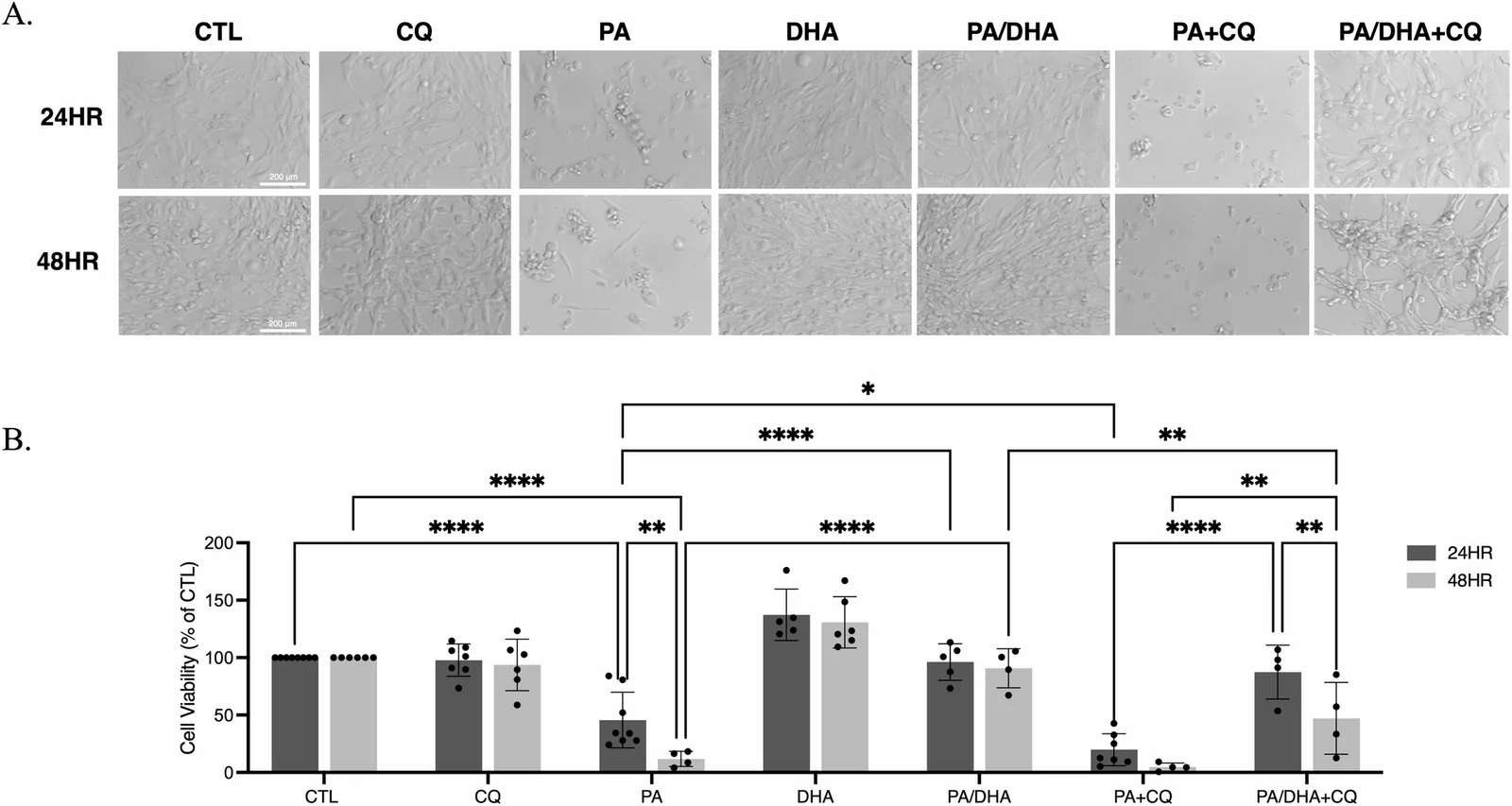
Functional Autophagy Supports Schwann Cell Viability During PA-Induced Lipotoxicity and Is Required for DHA Sustained Protection. ISC cultures were treated with 300 μM PA to induce lipotoxicity for 24 and 48 h, and with 50 μM DHA co-treatment to prevent cellular injury. To evaluate the role of autophagy flux, cells were co-treated with 20 μM chloroquine (CQ). (**A**) Representative phase-contrast images showing ISC morphology at 24- and 48-h post-treatments. (**B**) Cell viability assessed using WST-1 assay. Data are presented as mean ± SD (*N* > 4) and expressed as percent of control. Statistical significance was determined using a two-Way ANOVA followed by Holm–Šidák post hoc pairwise comparisons. *p < 0.05, **p < 0.01, ****p < 0.0001

Consistent with prior findings, PA significantly reduced cell viability, whereas co-treatment with DHA fully preserved ISC viability and spindle-shaped morphology (Fig. 1A, B). Treatment with CQ alone did not alter cell viability; however, the addition of CQ significantly exacerbated PA-induced cell death at 24 h (Fig. 1B). Notably, in the presence of CQ, DHA’s sustained protective effect against PA-LTx was significantly attenuated by 48 h (Fig. 1B). These findings demonstrate that pharmacological disruption of autophagic flux with CQ intensifies PA-mediated cytotoxicity and compromises DHA’s neuroprotective capacity. Together, the results suggest that intact autophagic flux serves as a key pro-survival mechanism during PA-LTx and is at least partially required for DHA’s protective actions in our ISC model.

### DHA Restores Autophagic Flux Impaired by Palmitic Acid-Induced Lipotoxicity

**Fig. 2:**
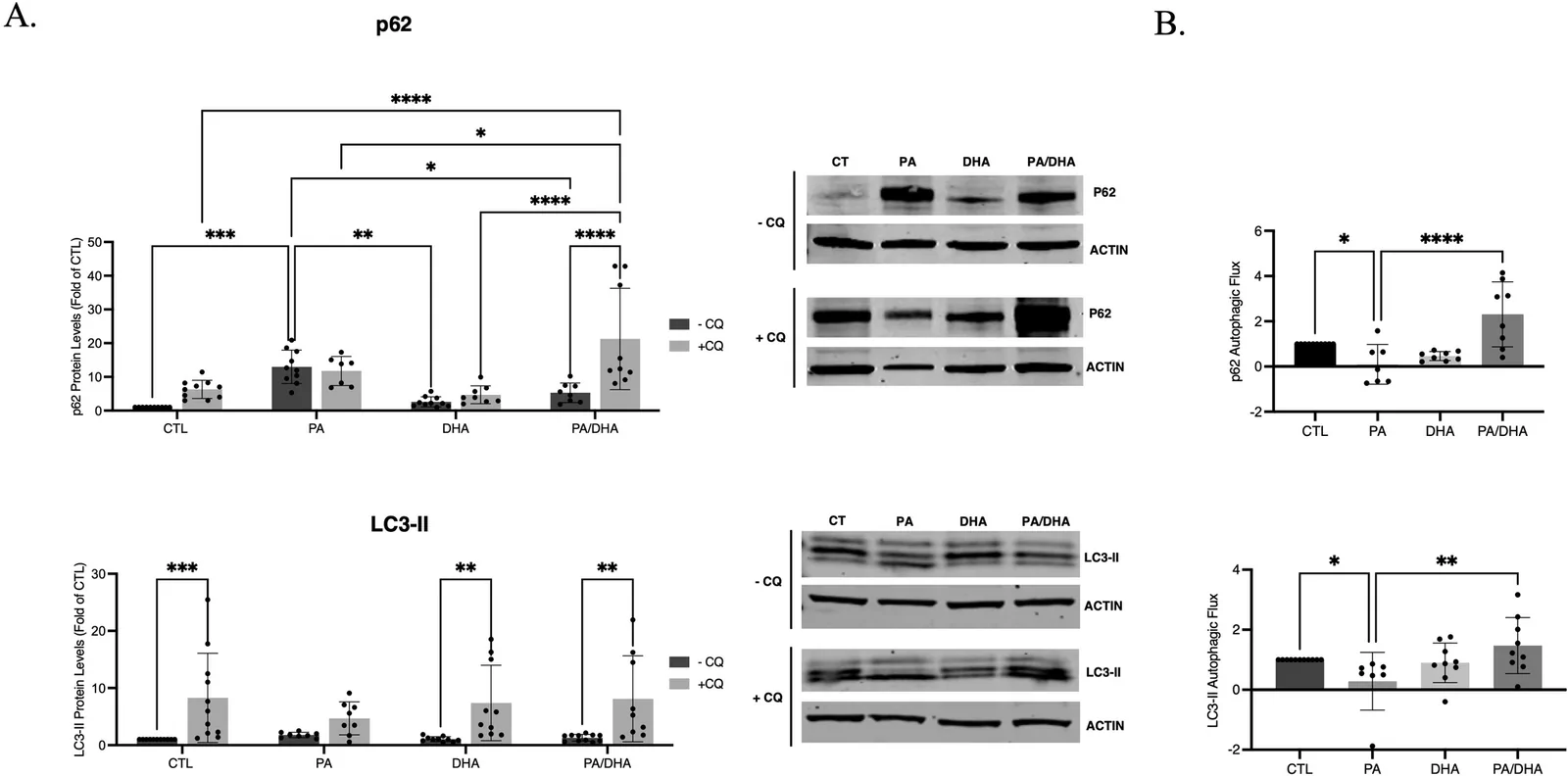
DHA Restores Autophagy Flux Disrupted by PA-LTx. ISCs were treated with 300 μM PA, 50 μM DHA, or a co-treatment of PA/DHA for 24 h. To assess autophagic flux, cells were co-treated with or without 20 μM chloroquine (CQ). (**A**) Representative Western blots showing protein levels of LC3-II and SQSTM1/p62, normalized to loading control β-actin. Protein band intensity quantification is presented as fold-change relative to control. (**B**) Autophagy flux was calculated as the difference in LC3-II and p62 between CQ-treated and untreated conditions for each fatty acid treatment, relative to the control. Data are presented as mean ± SD from *N* > 6 independent experiments. Statistical significance was determined by one-way or two-way ANOVA, followed by Holm-Šidák post hoc pairwise comparisons, as appropriate. *p < 0.05, **p < 0.01, ***p < 0.001, ****p < 0.0001

To determine the effect of PA-LTx and DHA co-treatment on autophagy dynamics in ISCs, protein levels of lipidated microtubule-associated protein 1 light chain 3 (LC3-II) and SQSTM1/p62 were quantified using Western blotting. LC3-II is a key marker of autophagosome formation, whereas p62 levels reflect autophagosome cargo degradation, making these proteins a valuable tool for assessing autophagy activity. PA treatment significantly increased LC3-II and p62 levels (Fig. 2A), indicative of impaired autophagic degradation. In contrast, DHA co-treatment markedly stabilized LC3-II and p62 levels towards those of untreated controls (Fig. 2A), consistent with preservation of autophagic clearance.

To directly assess autophagy flux, ISCs were treated with each lipid condition in the presence of flux inhibitor, chloroquine. In the presence of CQ, PA treatment showed no significant further accumulation of LC3-II and p62 relative to PA alone. Conversely, PA + DHA co-treatment in the presence of CQ resulted in significantly higher LC3-II and p62 levels compared to PA + DHA without CQ.

Autophagy flux was calculated as the difference in LC3-II and p62 levels between CQ-treated and untreated conditions for each fatty acid treatment. This analysis confirmed that PA significantly reduced autophagic flux, while DHA co-treatment restored flux towards control levels (Fig. 2B).

### DHA Maintains Autophagosome-Autolysosome Equilibrium During PA-Induced Lipotoxic Stress

To investigate the effects of PA-LTx and DHA co-treatment on autophagosome formation and autolysosome turnover, ISCs were transfected with an RFP-GFP LC3 tandem fluorescent reporter and imaged by confocal microscopy. The fraction of yellow (RFP + GFP+) and red-only (RFP + GFP-) puncta was quantified to determine shifts in autophagosome and autolysosome distribution in our different fatty acid treatments.

**Fig. 3:**
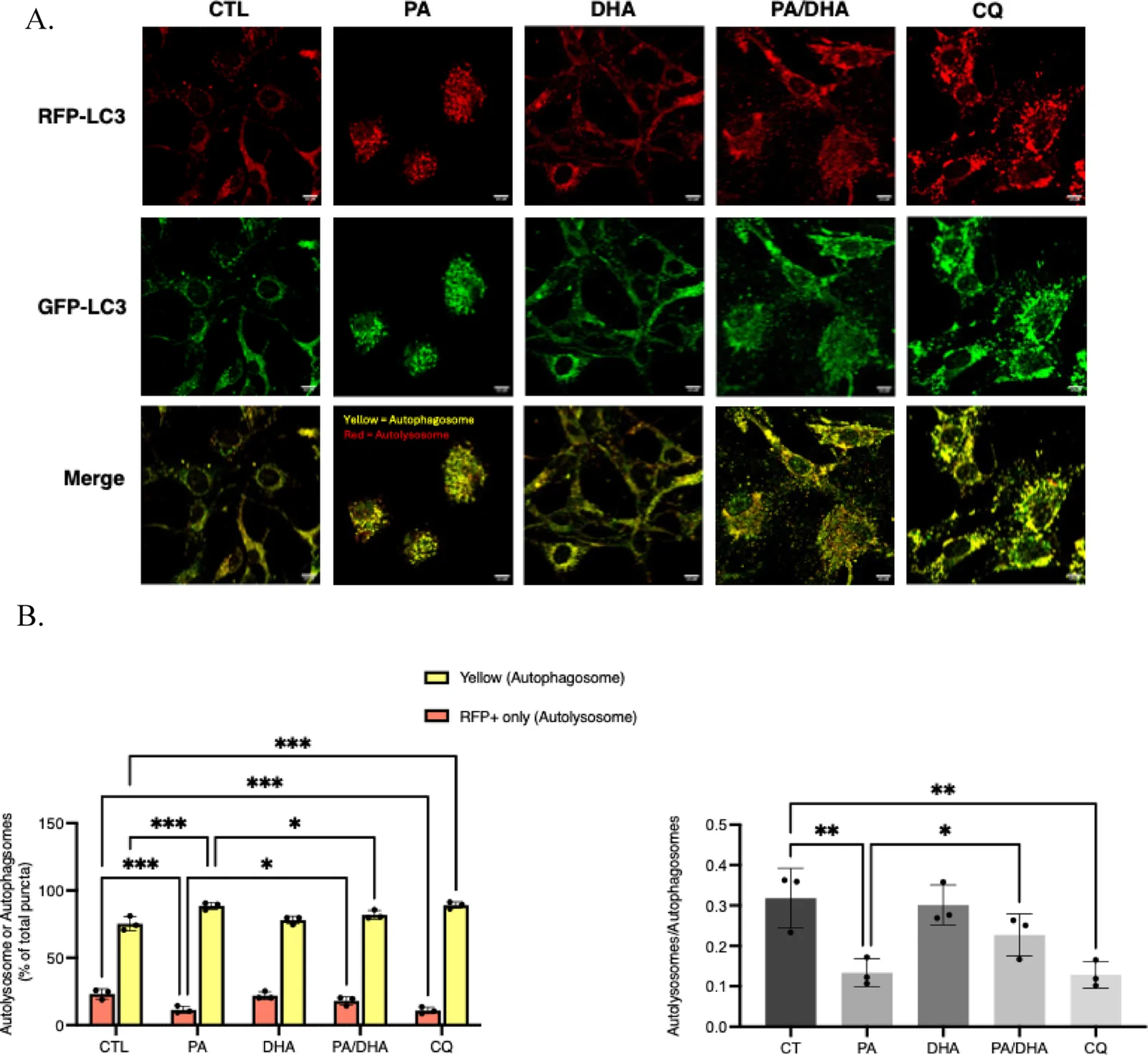
DHA Restores Autophagosome-Autolysosome Maturation During Palmitic Acid-Induced Lipotoxicity. ISCs were transfected with a tandem RFP-GFP-LC3 reporter construct to monitor autophagosome and autolysosome dynamics. Cells were treated with 300 µM PA, 50 µM DHA or PA + DHA for 24 h. Confocal images were acquired using a Zeiss LSM 900 microscope at 63x magnification (scale bar = 10 μm). **A** Representative confocal images showing RFP-GFP-LC3 puncta. Yellow puncta (GFP⁺RFP⁺) indicate autophagosomes prior to lysosomal fusion, whereas red-only puncta (RFP⁺) represent autolysosomes, where GFP fluorescence is quenched by the acidic lysosomal environment. **B** Quantification of puncta using Fiji/ImageJ. The number of yellow (autophagosomes), red+ only (autolysosomes), and total puncta was counted and normalized to cell area. The bar graph represents the yellow fraction (yellow puncta/total puncta) and red-only fraction (red-only puncta/total puncta) under each treatment condition. The ratio of the yellow (autophagosomes) and red-only fractions (autolysosomes) served as an index of autophagy flux. Data are presented as mean ± SD from *N* = 3 independent experiments, with 2–4 images analyzed for each treatment per experiment. Statistical significance was determined by one-way or two-way ANOVA, followed by Holm-Šidák post hoc pairwise comparisons, as appropriate. *p < 0.05, **p < 0.01, ***p < 0.00

PA treatment significantly increased the proportion of yellow puncta and decreased the proportion of red-only puncta relative to untreated controls (Fig. 3B), indicating an accumulation of autophagosomes and a reduction in autolysosome maturation. In contrast, DHA co-treatment restored the distribution of autophagosome (yellow puncta) and autolysosomes (red-only puncta) toward control levels (Fig. 3B), indicating that DHA preserves autophagosome–lysosomal fusion during lipotoxic stress. The ratio of autolysosome to autophagosome (red-only/yellow puncta fractions), used as a measure of autophagic flux, was significantly decreased by PA treatment but normalized by DHA co-treatment (Fig. 3B).

### DHA Attenuates PA-LTx-Induced ER Stress and Calcium Dysregulation

**Fig. 4:**
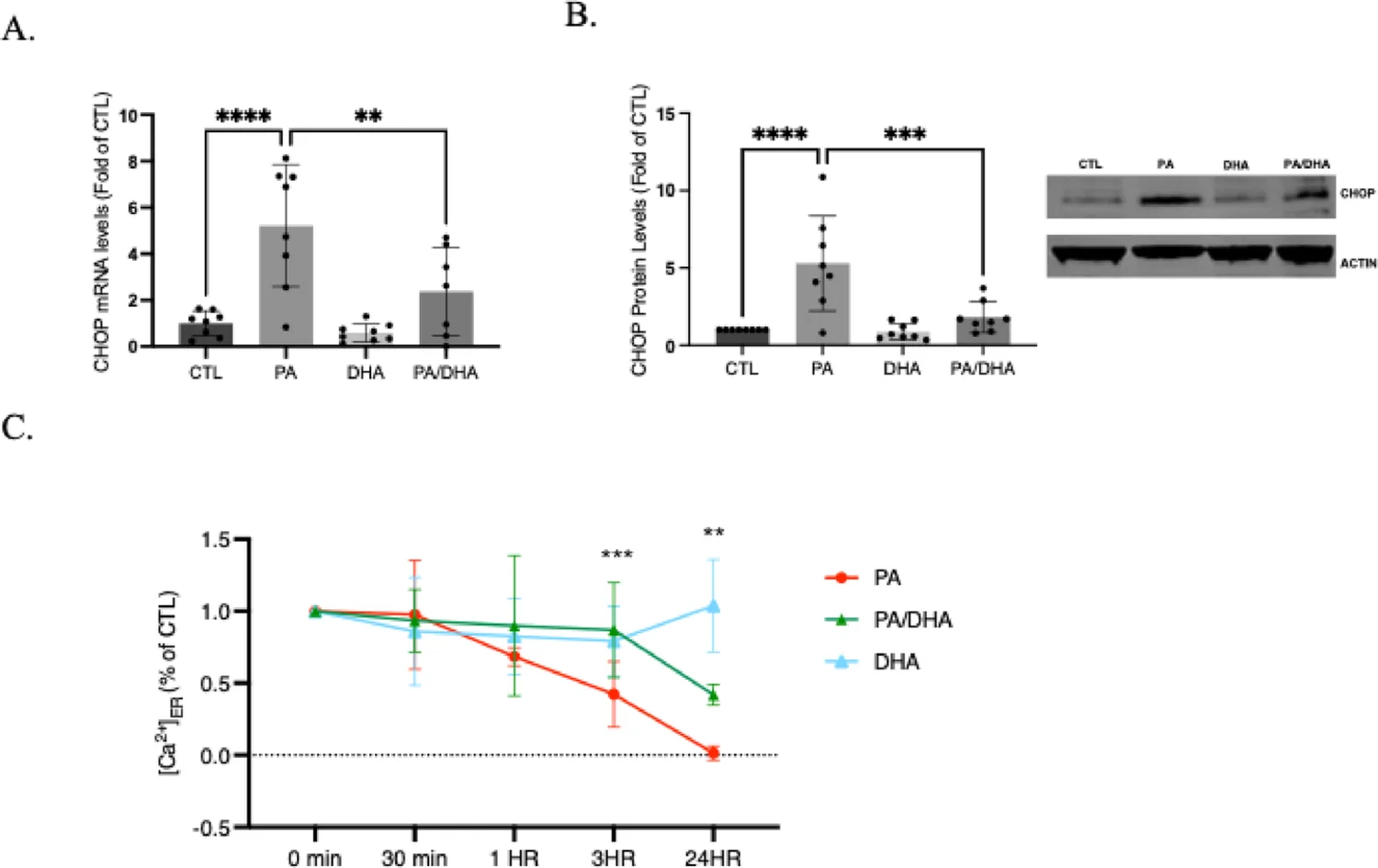
DHA Attenuated Palmitic Acid-Induced ER Stress Response and Calcium Dysregulation. ISCs were treated with 300 µM PA, 50 µM DHA, or PA + DHA for the indicated times. **A** CHOP mRNA expression was assessed using Real-time qPCR at 24 h post-treatments. **B** CHOP protein levels were evaluated using Western blot at 24 h post-treatments. Representative Western blot is shown, with β-actin as a loading control. **C** ER calcium release was measured using the Fluo-4 NW calcium assay at 30 min, 1 h, 3 h, and 24 h post-treatments. Data are presented as mean ± SD fold-change relative to control from *N* > 5 independent experiments. Statistical significance was determined by one-way or two-way ANOVA, followed by Holm-Šidák post hoc pairwise comparisons, as appropriate. For panel C, significance symbols indicate comparisons between PA vs. PA + DHA at each time point. *p < 0.05, **p < 0.01, ***p < 0.001, ****p < 0.0001

To investigate whether DHA-mediated protection against PA-LTx involves modulation of ER stress signaling and downstream calcium handling, expression of key ER stress markers was measured using real-time qPCR and Western blotting, and ER calcium release was evaluated using a Fluo-4 NW calcium assay. As anticipated, PA treatment markedly upregulated CHOP mRNA and protein levels and induced a time-dependent depletion of ER calcium stores (Fig. 4). In contrast, DHA co-treatment significantly attenuated CHOP induction and reduced the extent of ER calcium depletion caused by lipotoxic stress (Fig. 4). Comparable patterns were observed for additional unfolded protein response markers ATF4 and Xbp1, where PA activated these pathways, and DHA co-treatment counteracted their upregulation (Supplementary Figure S2).

### FABP5 Expression is Regulated by Palmitic Acid-Induced Lipotoxicity and Autophagy Modulation

**Fig. 5:**
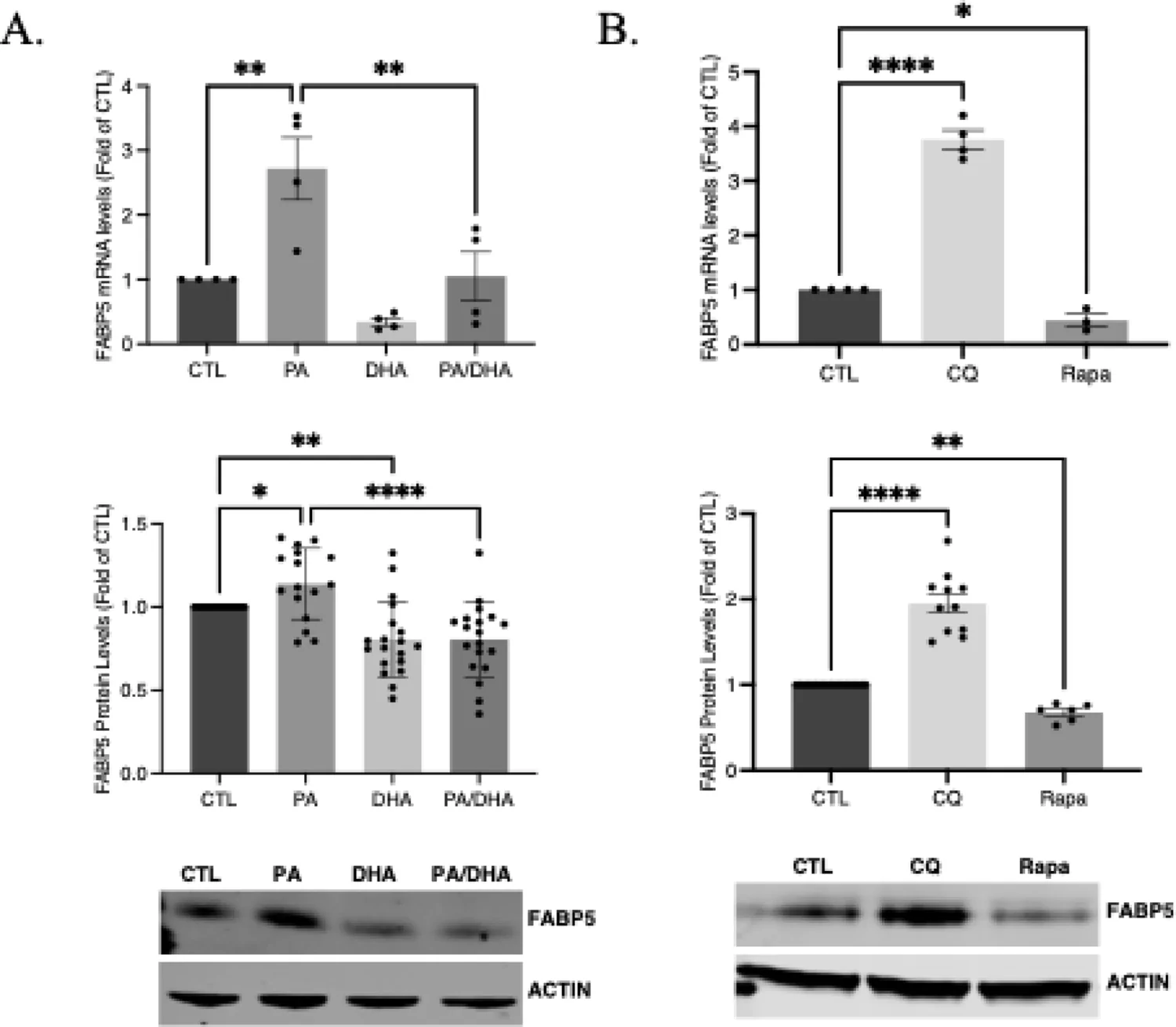
FABP5 expression in Response to Fatty Acids and Autophagy Modulation. ISCs were treated with 300 µM PA, 50 µM DHA, PA + DHA, 20 µM CQ, or 100 nM rapamycin for 24 h. FABP5 mRNA and protein levels were quantified by real-time qPCR and Western blotting, respectively. **A** FABP5 expression in response to fatty acid treatments. **B** FABP5 expression following treatments with the autophagy inhibitor Chloroquine or the autophagy inducer Rapamycin. Representative Western blot shown for FAPB5 protein in both panels, with β-actin as loading control. Data are presented as mean ± SD fold-change relative to control from *N* > 4 independent experiments. Statistical significance was determined by one-way ANOVA, followed by Holm-Šidák post hoc planned pairwise comparisons. *p < 0.05, **p < 0.01, ****p < 0.0001

FABP5 has been identified as a stress-responsive protein in neural injury models; however, its expression and regulation in Schwann cells have not been previously characterized. Therefore, FABP5 expression was evaluated following PA-induced lipotoxicity, DHA treatment, and pharmacological modulation of autophagy at 24 h using Western blot and real-time qPCR analyses. PA treatment significantly upregulated both FABP5 mRNA and protein expression compared to untreated controls. However, DHA co-treatment restored expression to control levels during lipotoxic stress (Fig. 5A). Notably, autophagy flux inhibitor Chloroquine markedly increased FABP5 mRNA and protein levels, whereas autophagy inducer Rapamycin reduced FABP5 expression compared to control (Fig. 5B).

### Silencing of FABP5 Alters Cell Viability and Stress Response Signaling Pathways During PA-Induced Lipotoxicity

**Fig. 6:**
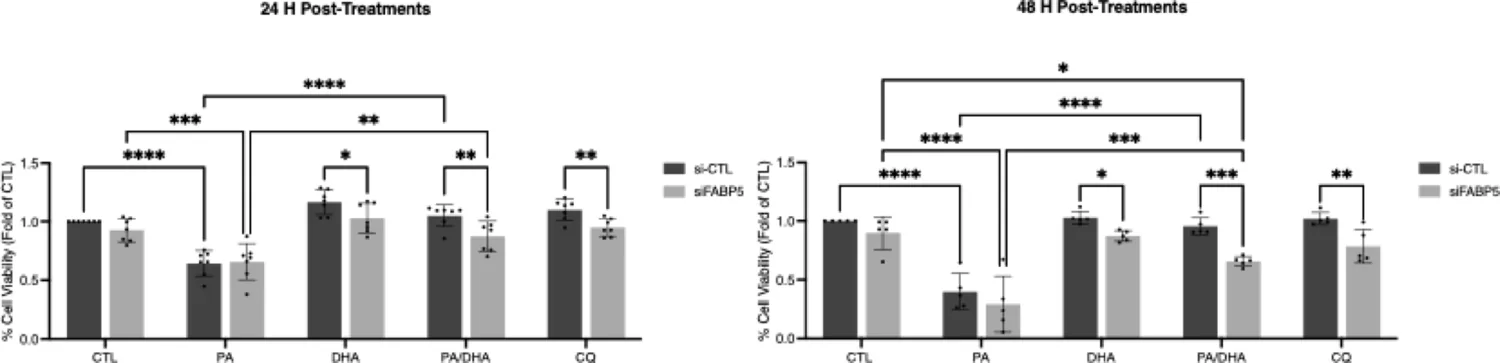
FABP5 knockdown Attenuates DHA-mediated Protection. ISCs were transfected with FABP5-targeting siRNA (si-FABP5) or scramble control siRNA (si-CTL) for 24 h, followed by fatty acid and CQ treatments for an additional 24–48 h. Cell viability assessed using WST-1 assay. Data are presented as mean ± SD (*N* > 5) and expressed as percent of control. Statistical significance was determined using a two-Way ANOVA followed by Holm–Šidák post hoc pairwise comparisons. *p < 0.05, **p < 0.01, ****p < 0.0001

To identify the functional role of FABP5 in cellular adaptation responses to lipotoxic stress and its contribution to DHA-mediated protection, ISCs were transfected with either scramble (control) siRNA or FABP5-targeting siRNA. Efficient knockdown of FABP5 expression (> 70%) was confirmed by mRNA and protein levels 24 h post-transfection (Supplementary Figure S3). Expression of other FABP family members was also quantified by real-time quantitative PCR and showed no compensatory upregulation or significant changes upon FABP5 silencing (Supplementary Figure S4). Transfected cells were then treated with 300 µM PA, 50 µM DHA, or PA + DHA for an additional 24 h. Cell viability was assessed using the WST-1 assay at 24 h and 48 h following these fatty acid treatments in si-CTL and si-FABP5 cells (Fig. 6). FABP5 silencing significantly reduced viability under DHA, PA + DHA, and CQ conditions at both time points. Although a reduction in viability was also observed in the CTL and PA groups at 48 h, this effect did not reach statistical significance.

**Fig. 7:**
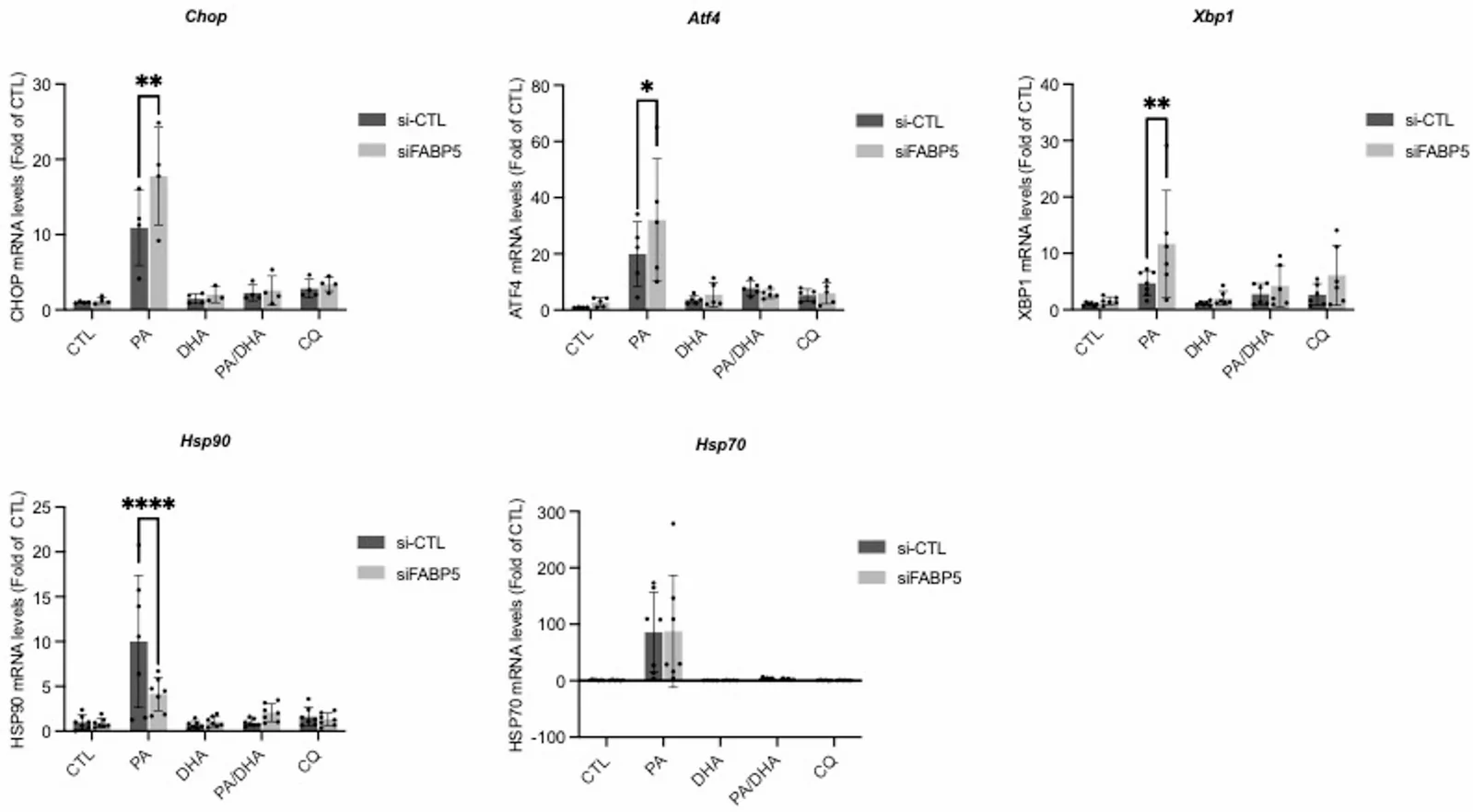
FABP5 Knockdown in ISCs Modulates Expression of ER stress-Associated Genes During PA-Induced Lipotoxicity. ISCs were transfected with FABP5-targeting siRNA (si-FABP5) or scramble control siRNA (si-CTL) for 24 h, followed by fatty acid and CQ treatments for an additional 24 h. mRNA expression of selected ER stress/UPR genes (Xbp1, CHOP, ATF4, Hsp90, and Hsp70) were quantified by real-time qPCR. Data are presented as mean ± SD fold-change relative to si-CTL untreated control from *N* > 5 independent experiments. Statistical significance was determined by two-way ANOVA followed by Holm-Šidák post hoc pairwise comparisons. *p < 0.05, **p < 0.01, ***p < 0.001, ****p < 0.0001

First, we examined the effect of FABP5 loss on ER stress in ISCs by measuring mRNA expression of key ER stress markers and heat shock proteins. In PA-treated cells, FABP5 silencing upregulated mRNA expression of ER stress-related genes Xbp1, CHOP, and ATF4, compared to scramble siRNA PA-treated cells (Fig. 7) significantly. Additionally, FABP5-deficient cells showed a selective reduction in Hsp90 mRNA expression during PA exposure, whereas Hsp70 mRNA expression remained unchanged (Fig. 7). These findings suggest that FABP5 normally attenuates ER stress during lipotoxic conditions and selectively supports aspects of the heat shock response.

**Fig. 8:**
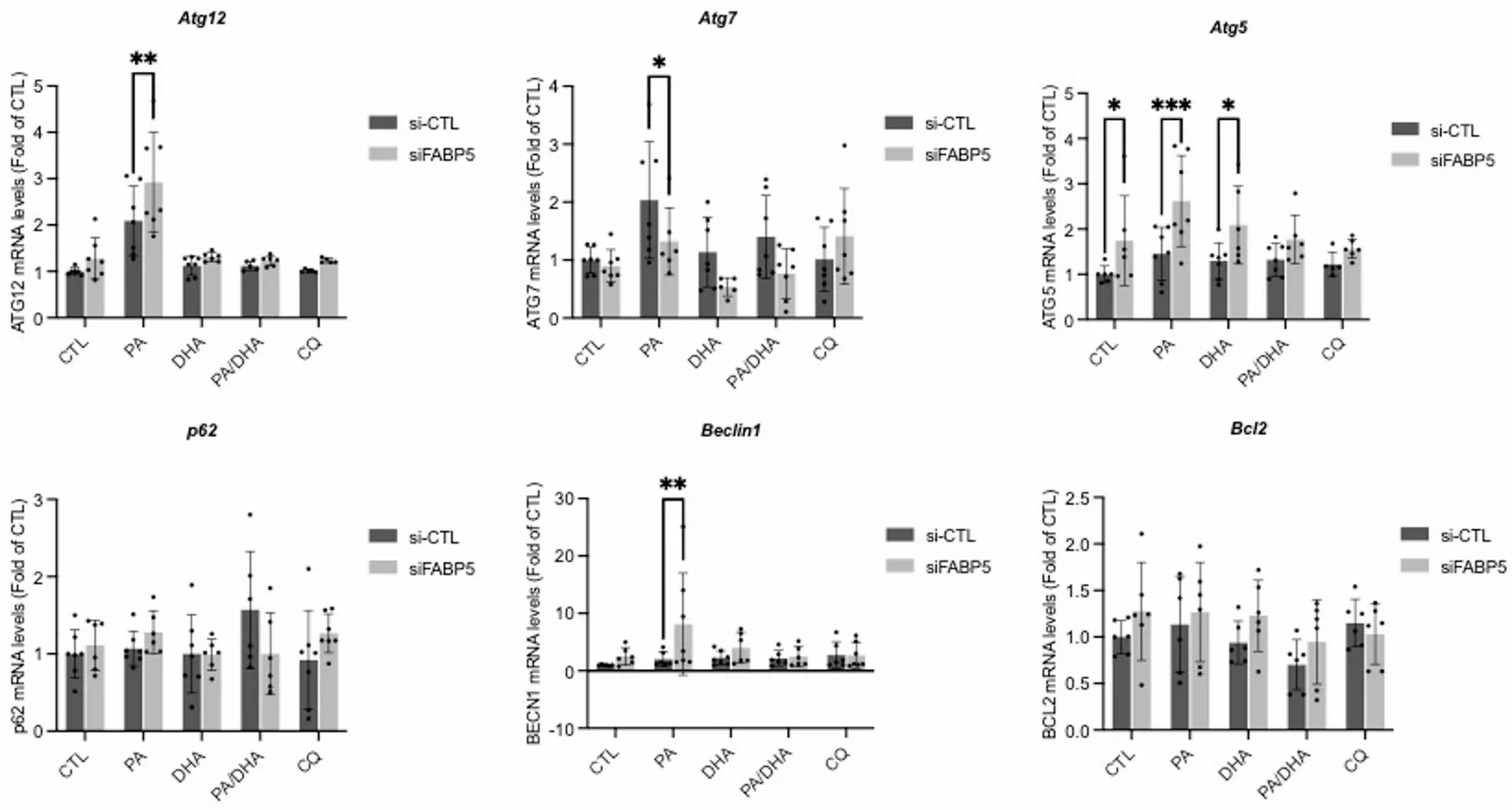
FABP5 Knockdown in ISCs Modulates Expression of Autophagy-Related Genes During PA-Induced Lipotoxicity. ISCs were transfected with FABP5-targeting siRNA (si-FABP5) or scramble control siRNA (si-CTL) for 24 h, followed by fatty acid and CQ treatments for an additional 24 h. mRNA expression of selected autophagy -related genes (Beclin-1, ATG5, ATG12, ATG7, p62 (SQSTM1), and BCL2) were quantified by real-time qPCR. Data are presented as mean ± SD fold-change relative to si-CTL untreated control from *N* > 5 independent experiments. Statistical significance was determined by two-way ANOVA followed by Holm-Šidák post hoc pairwise comparisons. *p < 0.05, **p < 0.01, ***p < 0.001, ****p < 0.0001

Next, because FABP5 is a lipid chaperone involved in intracellular fatty acid trafficking and autophagosome biogenesis requires extensive lipid membrane remodeling, we next examined whether FABP5 modulates the transcriptional response of autophagy-related genes. In PA-treated cells, FABP5 knockdown significantly increased mRNA levels of autophagy initiation and membrane formation genes Beclin-1, ATG5, and ATG12, while decreasing ATG7 expression. ATG5 mRNA was also significantly elevated in untreated control and DHA-treated cells upon FABP5 knockdown. The mRNA levels of p62 and BCL2 were unchanged across all conditions (Fig. 8). These results indicate that loss of FABP5 induces a compensatory transcriptional upregulation of early autophagy components but suppresses ATG7, an E1-like enzyme essential for LC3 lipidation and autophagosome maturation.

**Fig. 9:**
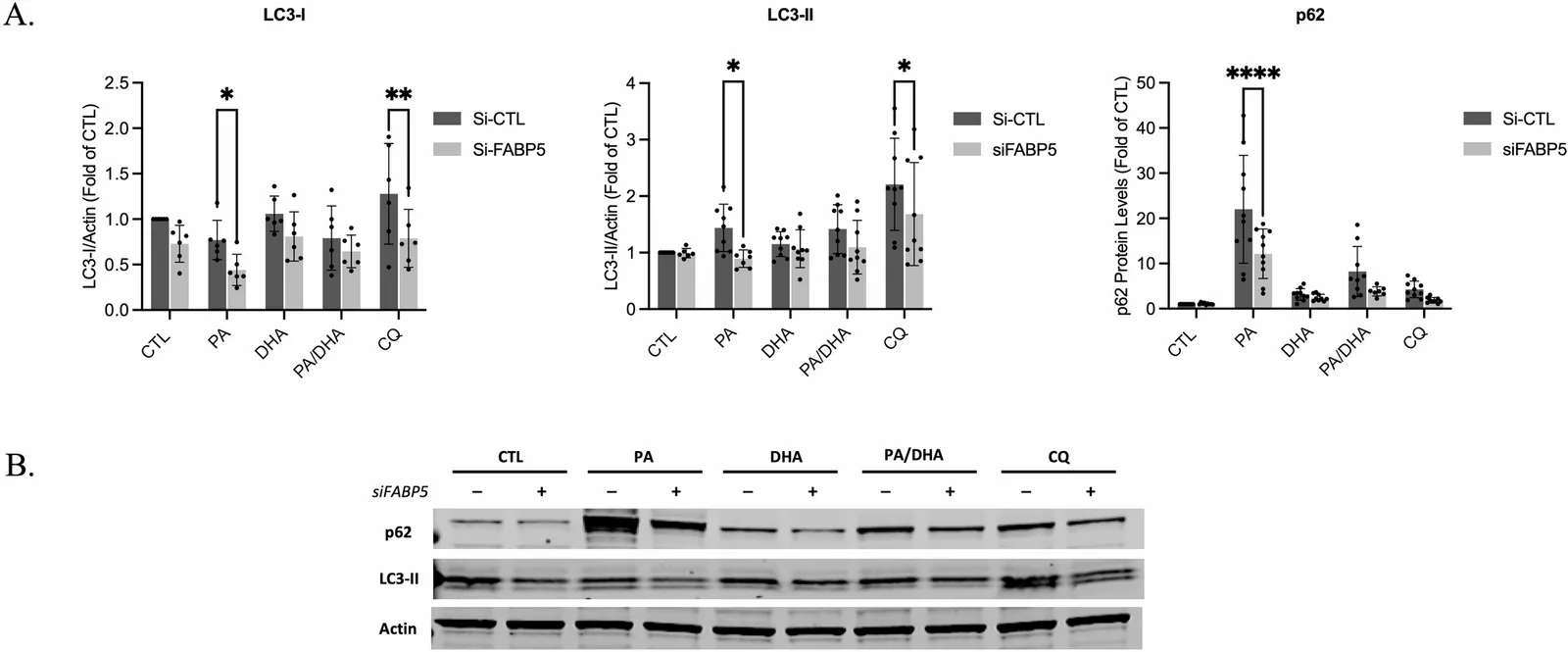
FABP5 Knockdown Effect on Autophagosome Formation and Degradation. Autophagosome formation and degradation was assessed in ISCs exposed to 24 h fatty acid treatments following FABP5 knockdown. Protein levels of LC3 I, LC3-II, and p62 were quantified using Western blotting and normalized to β-actin. **A** Quantification of LC3-I, LC3-II, and p62 protein levels. Data are presented as mean ± SD fold-change relative to si-CTL untreated control from *N* > 6 independent experiments. **B** Representative blots for LC3 (showing LC3-I and LC3-II) and p62 under the indicated conditions are shown. Statistical significance was determined by two-way ANOVA, followed by Holm-Šidák post hoc pairwise comparisons. *p < 0.05, ***p < 0.001

To determine whether the observed transcriptional changes in autophagy genes following FABP5 silencing translated to functional alterations in autophagy flux, LC3 and p62 protein levels were quantified by Western blotting. Despite elevated expression of autophagy initiation genes, FABP5 silencing reduced PA-induced accumulation of LC3-II and p62 compared to PA-treated scramble siRNA controls. Moreover, the robust accumulation of LC3-II and p62 normally induced by CQ was markedly attenuated in FABP5-deficient cells (Fig. 9). LC3-I levels, which reflect the available LC3 pool, were significantly decreased in PA- and CQ-treated FABP5-knockdown cells (Fig. 9). Collectively, these results demonstrate that FABP5 silencing results in autophagy defects upstream of lysosomal degradation, likely due to reduced LC3 availability and defective autophagosome formation, rather than enhanced flux.

### FABP5 Silencing Suppresses DHA-Induced Lipid Droplet Formation

**Fig. 10:**
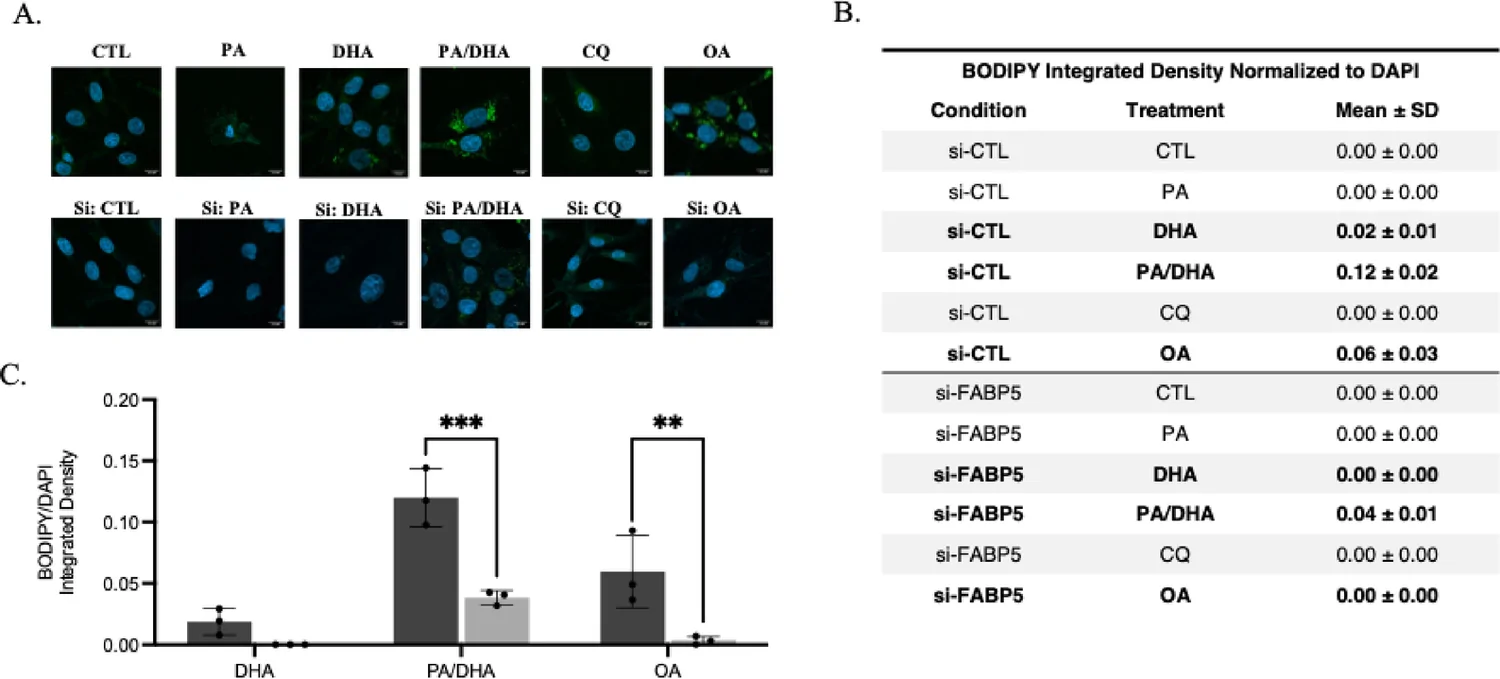
FABP5 Silencing Suppresses Lipid Droplet Formation. Following siRNA-mediated knockdown of FABP5 and subsequent 24-h fatty acid treatments, ISCs were fixed and stained with 10 µM BODIPY 493/503 to visualize and quantify lipid droplet abundance. Images were acquired on a Zeiss LSM 900 confocal microscope at 63x magnification (scale bar = 10 μm). Oleic acid (OA) served as the positive control for lipid droplet induction. **A** Representative fluorescence images showing BODIPY 493/503 in si-CTL and si-FABP5 cells under the indicated treatments. **B** Quantification of lipid droplet abundance. BODIPY 493/503 integrated density was measured using Fiji/ImageJ and normalized to DAPI intensity. Mean ± SD values for each condition are reported in the table from *N* = 3 independent experiments; 2–4 images per treatment group analyzed per independent experiment. **C** Treatments showing induction of lipid droplets were analyzed using two-way ANOVA, followed by Holm-Šidák post hoc pairwise comparisons between si-CTL and si-FABP5 groups. *p < 0.05, ***p < 0.001

Lipid droplets function as adaptive buffering organelles that sequester excess fatty acids and help maintain ER homeostasis and autophagic competence [24]. Because FABP5 is involved cellular lipid remodeling and loss of FABP5 altered ER stress and autophagy signaling, lipid droplet formation was next evaluated in FABP5-deficient ISCs following fatty acid treatments using BODIPY 493/503 fluorescence staining. In non-silenced cells, lipid droplets were not detected under control, PA, and CQ treatments. In contrast, ISCs treated with DHA and PA/DHA co-treatment had significant lipid droplet accumulation, with OA serving as the positive control for lipid droplet induction (Fig. 10A, B). These findings indicate that DHA promotes lipid droplet formation during PA-LTx. Relative to non-silencing controls, FABP5-silenced ISCs had a marked reduction in BODIPY 493/503 integrated density in DHA, PA/DHA, and OA treatments (Fig. 10C). These results demonstrate that FABP5 is required for efficient lipid droplet formation and neutral lipid sequestration in response to fatty acid treatments that drive lipid storage, particularly under DHA-mediated protective conditions.

## Discussion

### Overview and Conceptual Advance

This study identifies preservation of autophagic flux as a central determinant of Schwann cell survival during lipotoxic stress and establishes FABP5 as a previously unrecognized coordinator of lipid trafficking, ER homeostasis, and autophagic competence in peripheral glia. Using an immortalized Schwann cell (ISC) model, we show that palmitic acid (PA) exposure induces ER stress and calcium imbalance, disrupts autophagic degradation, and fails to promote lipid droplet sequestration, culminating in cytotoxicity. In contrast, docosahexaenoic acid (DHA) counteracts these maladaptive responses by stabilizing ER–calcium homeostasis, restoring lysosomal clearance, and promoting neutral lipid storage, thereby sustaining Schwann cell function.

We demonstrate that DHA’s sustained cytoprotective effects depend on intact autophagic degradation rather than on autophagy induction alone. We further identify FABP5 as a stress-responsive node that links lipid handling to autophagic capacity, positioning it as an integrative regulator of metabolic stress adaptation in Schwann cells.

### Autophagic Flux, Not Autophagy Induction, Determines Schwann Cell Survival

Autophagy is a dynamic, multistep degradative pathway, and its functional output is defined by flux through the lysosomal compartment, rather than autophagosome accumulation [52]. Short-term PA exposure (< 12 h) can transiently activate autophagy as a protective response [53–55], whereas prolonged exposure disrupts autophagic clearance across multiple cell types [6, 27]. Our data extend these observations to Schwann cells and demonstrate that PA-induced cytotoxicity is exacerbated by pharmacologic inhibition of autophagic flux, directly implicating lysosomal dysfunction in lipotoxic injury.

Consistent with defective flux, prolonged PA exposure led to concurrent accumulation of LC3-II and SQSTM1/p62, without further accumulation in the presence of chloroquine — a hallmark of impaired autophagic degradation [52]. Tandem RFP-GFP-LC3 imaging corroborated these findings, revealing autophagosome accumulation with reduced autolysosome formation. These results demonstrate that autophagy initiation persists during PA stress but becomes uncoupled from effective degradation, rendering induction alone insufficient for Schwann cell survival.

**Fig. 11:**
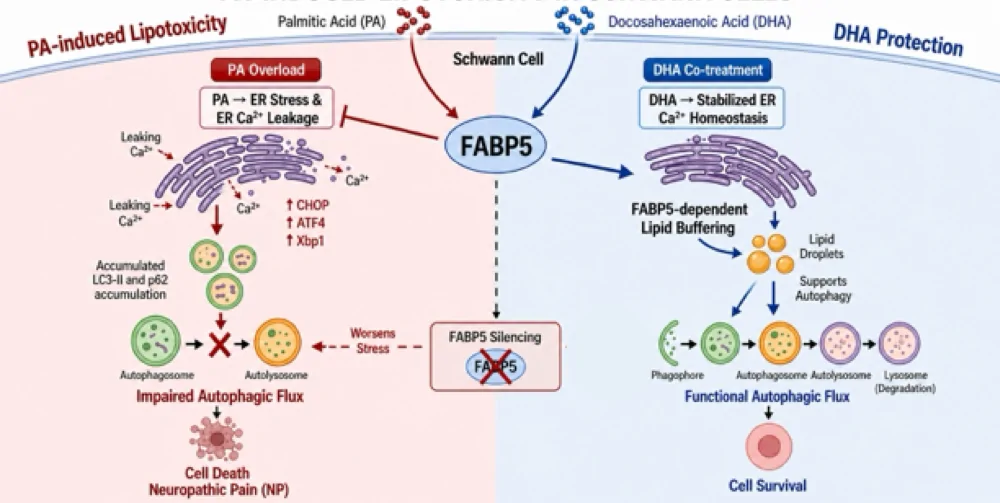
Schematic overview of the mechanisms underlying PA-induced lipotoxicity and the protective effects of DHA co-treatment in Schwann cells. Under PA overload (left, pink panel), ER stress and ER Ca²⁺ leakage can contribute to impaired autophagic flux. This is evidenced by PA-induced accumulation of LC3-II and p62 and disrupted autophagosome-lysosome fusion, ultimately leading to cell death that is commonly associated with neuropathic pain pathology. FABP5 silencing (center) exacerbates PA-induced lipotoxicity by further worsening ER stress and autophagic impairment, supporting a protective role for FABP5 in cellular stress adaptation. In contrast, DHA co-treatment (right, blue panel) stabilizes ER Ca²⁺ homeostasis and restores functional autophagic flux, while engaging FABP5 to promote adaptive lipid buffering, thereby supporting Schwann cell survival. Notably, DHA retains partial protective activity following FABP5 silencing, suggesting that DHA exerts both FABP5-dependent and FABP5-independent cytoprotective effects

### ER Stress and Calcium Dysregulation Constrain Autophagic Capacity

The endoplasmic reticulum (ER) is a central platform for autophagosome biogenesis, with phosphatidylinositol 3-phosphate-enriched ER subdomains serving as sites of phagophore nucleation [56, 57]. Consequently, PA-induced ER dysfunction is well positioned to limit autophagic capacity [28, 58]. Mechanistically, disruption of ER homeostasis and calcium handling has been closely linked to impaired autophagic flux during lipotoxic stress [29, 59].

In astrocytes, prolonged PA exposure induces CHOP expression, impairs autophagic clearance, and promotes inflammatory signaling [29]. In cortical neurons, acute ER stress activates PERK-eIF2α-ATF4 and IRE1-JNK signaling, transiently supporting autophagy initiation, whereas sustained stress leads to autophagy failure and apoptosis [59–62]. Beyond protein folding, PA inhibits SERCA pump activity, resulting in ER calcium depletion and sustained cytosolic calcium elevation—conditions known to impair Rab7-dependent autophagosome-lysosome fusion [63, 64].

In line with this framework, PA-treated ISCs exhibited induction of CHOP, ATF4, and Xbp1, accompanied by time-dependent depletion of ER calcium stores and coincident loss of autophagic flux. These findings position ER dysfunction as an upstream constraint on autophagy execution, rather than a downstream consequence of impaired degradation.

### DHA Preserves Late-Stage Autophagy by Stabilizing ER Homeostasis

Consistent with prior work from our group [15, 16], DHA robustly preserved Schwann cell viability during prolonged PA exposure. However, this protection was significantly attenuated by chloroquine, demonstrating that DHA’s cytoprotective effects depend on functional autophagic degradation. Notably, DHA normalized LC3-II and p62 dynamics and restored their accumulation in the presence of chloroquine, indicating preservation of autophagic flux rather than suppression of autophagy signaling. These findings align with studies showing that DHA modulates mTOR-dependent autophagy pathways and prevents PA-induced apoptosis [16, 65]. In Schwann cells exposed to oxidative stress, DHA similarly restores autophagy dynamics through AMPK/ULK1 signaling [66].

Importantly, our data shows that DHA selectively preserves late-stage autophagy, maintaining autophagosome–lysosome fusion rather than merely increasing autophagosome formation. Parallel suppression of PA-induced ER stress markers and stabilization of calcium handling further suggest that DHA maintains autophagic competence by preserving ER homeostasis [67–69]. Together, these results refine existing models by demonstrating that DHA-mediated protection depends on intact lysosomal clearance, a requirement for sustained cellular adaptation to lipid overload.

### FABP5 Integrates Lipid Stress Sensing with Autophagic Competence

A key novel finding of this study is the identification of FABP5 as a regulator of lipid stress adaptation and autophagy competence in Schwann cells. PA robustly induced FABP5 expression, consistent with its role as a stress-responsive lipid chaperone activated by oxidative stress, lipotoxicity, ferroptosis, and hypoxia [43, 70, 71]. FABP5 binds both saturated and unsaturated long-chain fatty acids, with functional outcomes determined by ligand context [49, 72].

DHA co-treatment normalized PA-induced FABP5 upregulation, suggesting that DHA alleviates upstream stress signaling and reduces reliance on FABP5-mediated lipid buffering. Autophagy modulation independently regulated FABP5 expression: chloroquine robustly increased FABP5 levels, whereas rapamycin reduced them. Given chloroquine’s known induction of phospholipidosis [73, 74], FABP5 induction likely reflects compensatory responses to lysosomal lipid accumulation, further linking FABP5 to lipid stress management.

FABP5 silencing exacerbated PA-induced ER stress, selectively reduced HSP90 abundance, and disrupted coordinated regulation of autophagy machinery. Although BECN1, ATG5, and ATG12 were induced as part of a compensatory response [75], ATG7—essential for LC3 lipidation—was reduced [76, 77], indicating impaired execution of autophagy despite upstream activation. Mechanistically, FABP5 supports lipid delivery to the ER for membrane synthesis [78, 79] and regulates endocannabinoid trafficking to FAAH, generating ethanolamine required for phosphatidylethanolamine synthesis [80]. Given the requirement of phosphatidylethanolamine for LC3 conjugation, reduced LC3-II abundance following FABP5 silencing likely reflects limited lipid substrate availability for autophagosome biogenesis. Future studies using tandem RFP-GFP-LC3 imaging in FABP5-silencing and overexpression models would help further define how FABP5 regulates autophagic flux under lipotoxic stress.

### FABP5 Contributes to DHA-Induced Lipid Droplet Formation and Cytoprotection

Consistent with prior reports, PA failed to efficiently induce lipid droplet formation, whereas DHA robustly promoted neutral lipid sequestration [20, 21]. Interestingly, minimal lipid droplet accumulation was observed in control cells, likely reflecting the absence of excess fatty acid burden under basal conditions. Because lipid droplets primarily function as adaptive storage organelles during lipid stress, significant lipid droplet biogenesis would not be expected in untreated cells [81]. FABP5 silencing significantly impaired DHA-induced lipid droplet accumulation, suggesting that FABP5 contributes to lipid handling and efficient lipid droplet biogenesis during metabolic stress [48, 82].

Lipid droplets function not only as detoxification reservoirs for excess fatty acids, but also as lipid sources for autophagosome membrane formation at ER–lipid droplet contact sites [20, 23, 25]. Therefore, reduced lipid droplet accumulation during PA treatment and following FABP5 silencing may reflect impaired lipid-supported stress adaptation and autophagic capacity rather than an isolated defect in lipid storage alone.

Although FABP5 silencing significantly attenuated several aspects of DHA-mediated cytoprotection, DHA retained partial protective activity in FABP5-deficient cells. This indicates that DHA exerts FABP5-independent effects, potentially through direct modulation of membrane composition and fluidity, suppression of ER stress signaling, antioxidant activity, or activation of parallel lipid-handling and stress-adaptive pathways.

### Study Limitations

Despite the findings presented in this study, some limitations should be acknowledged. The present study primarily utilized immortalized Schwann cells (iSCs) under N-2-supplemented serum-free conditions to limit cell proliferation. In addition, chloroquine was used to assess autophagic flux; however, like many autophagy inhibitors, it can have off-target effects. Future work employing primary Schwann cells, in vivo models, or alternative flux assessment methods will therefore be important to further validate and extend our observations. Importantly, however, our previous study [15] demonstrated that DHA similarly reversed PA-induced apoptotic cell death in primary Schwann cells, supporting the translational relevance of the current findings.

### Implications for Metabolic Stress–Associated Neuropathies

Together, these findings define a lipotoxicity-driven cascade linking ER stress, autophagic failure, and defective lipid handling to Schwann cells, and identify DHA–FABP5 signaling as a key counter-regulatory axis (Fig. 11). By bridging clinical observations with cell-specific mechanisms, this work highlights lipid metabolism and autophagic competence as actionable targets in metabolic neuropathies. Strategies that preserve autophagic flux through dietary or pharmacologic modulation of FABP5-dependent lipid trafficking may represent a tractable approach to mitigating Schwann cell dysfunction and neuropathic pain.

## Data Availability

The datasets generated and/or analyzed during the current study are available from the corresponding author upon reasonable request.
